# G protein-coupled receptor kinase 2 (GRK2) as a multifunctional signaling hub

**DOI:** 10.1007/s00018-019-03274-3

**Published:** 2019-08-20

**Authors:** Petronila Penela, Catalina Ribas, Francisco Sánchez-Madrid, Federico Mayor

**Affiliations:** 1 Departamento de Biología Molecular, Centro de Biología Molecular “Severo Ochoa” (UAM-CSIC), Universidad Autónoma de Madrid, C/Nicolás Cabrera 1, 28049 Madrid, Spain; 2 Instituto de Investigación Sanitaria La Princesa, 28006 Madrid, Spain; 3 CIBER de Enfermedades Cardiovasculares, ISCIII (CIBERCV), 28029 Madrid, Spain; 4 Cell-Cell Communication Laboratory, Vascular Pathophysiology Area, Centro Nacional Investigaciones Cardiovasculares (CNIC), 28029 Madrid, Spain

**Keywords:** GRK2, GPCR, Phosphorylation, Interactome, HDAC6

## Abstract

Accumulating evidence indicates that G protein-coupled receptor kinase 2 (GRK2) is a versatile protein that acts as a signaling hub by modulating G protein-coupled receptor (GPCR) signaling and also via phosphorylation or scaffolding interactions with an extensive number of non-GPCR cellular partners. GRK2 multifunctionality arises from its multidomain structure and from complex mechanisms of regulation of its expression levels, activity, and localization within the cell, what allows the precise spatio-temporal shaping of GRK2 targets. A better understanding of the GRK2 interactome and its modulation mechanisms is helping to identify the GRK2-interacting proteins and its substrates involved in the participation of this kinase in different cellular processes and pathophysiological contexts.

## Introduction

G protein-coupled receptor kinases (GRKs) constitute a family of seven serine/threonine protein kinases that specifically phosphorylate agonist-activated G protein-coupled receptors (GPCRs). Receptor phosphorylation triggers the binding of cytoplasmic β-arrestin molecules, which sterically block interaction with heterotrimeric G proteins, leading to rapid desensitization of G protein-mediated signaling cascades. Moreover, β-arrestins-bound GPCRs are targeted for clathrin-mediated endocytosis, a process that serves to de-phosphorylate, re-sensitize, and eventually recycle receptors back to the plasma membrane [1–5]. Beyond this canonical role in GPCRs desensitization, GRKs are instrumental in triggering G protein-independent GPCR signaling through β-arrestins, since the latter are scaffold proteins for many cellular partners, able to assemble complex GPCR signalosomes. Both the ligand-induced conformational changes and the phosphorylation pattern (called phosphorylation barcoding) imprinted by GRKs on GPCRs could be important for determining the balance between G protein activation, GPCRs desensitization, and GRK/β-arrestin-mediated signaling [6–10].

On the other hand, it is worth noting that the impact of changes in GRK expression and functionality in cells might also involve non-GPCR targets. Thus, GRKs and, in particular, the ubiquitous and essential GRK2 isoform, are emerging as signal transducers by themselves, being able to interact with (and in many cases, phosphorylate) a variety of non-GPCR proteins [2, 5, 11–14] see also below). Consistent with such complex interactomes, GRK2 has been reported to participate in many cellular and physiological processes (cardiac contractility, cell proliferation, cell cycle regulation, angiogenesis, vasodilatation, cell migration, inflammation and metabolic homeostasis, to name some relevant examples). The changes in GRK2 levels and functionality taking place in different pathological contexts were suggested to play a key role in disease progression [11, 15–20].

Therefore, a main current challenge is to elucidate the GPCR and/or non-GPCR GRK2 cellular partners that are involved in the physiological and pathological roles of this kinase and to better understand how GRK2 interaction networks are engaged in a stimulus-, cell type-, or context-specific manner. Such GRK2 multifunctionality appears to arise from its multidomain structure and also from complex mechanisms of regulation that determine GRK2 expression levels, activity, localization within the cell, and partner/target selection.

## An overview of the complexity of the GRK2 interactome

In addition to its canonical role in agonist-stimulated GPCR phosphorylation, GRK2 has been reported to establish functional or scaffolding interactions with an extensive and increasing number of cellular partners (reviewed in [2, 11, 12, 21]. Alternative GRK2 substrates and/or interactors include non-GPCR membrane receptors and proteins (such as PDGF or EGF receptor tyrosine kinases or the ENaC sodium channel), key cellular kinases (p38Mapk, AMPK, PI3K/AKT, MEK1, MST1), signal transducers and switchers (IRS1, RKIP, EPAC1, Pin1, Patched, PDEγ, eNOS, APC, Gαq, Gβγ, phosducin; RhoA, RalA), transcription factors and modulators (Smad2/3, Period1/2, IκBα, DREAM), E3 ubiquitin ligases and chaperones (Mdm2, Cul4A-DDB1-Gβ, Nedd4-2, Hsp90), ribosomal proteins, membrane components (clathrin, caveolin), mitochondrial proteins (Mitofusin1/2) or cytoskeleton components, and regulators (HDAC6, ezrin/radixin, tubulin, GIT1, α-actinin) (see Table 1 for the references and description of these GRK2 partners).

**Table 1:** GRK2 regulatory and effector interactome

Non-GPCR GRK2’s partners	Type of interaction	Regulatory consequence on		Cellular response and molecular impact	References
		GRK2	Partner		
Ligases and chaperones					
Cul4A-DDB1-Gβ	Direct interaction and ubiquitination of GRK2	Decreased protein stability		↓ GPCR receptor desensitization and anti-cardiac hypertrophy, anti-hypertension	[100, 188]
Nedd4-2	Direct phosphorylation of Nedd4-2		Unknown	ENAC channel stabilization?	[189]
Mdm2	Ubiquitination of GRK2	Decreased protein stability		↑ Classical βAR-G protein signaling ↓GPCR receptor desensitization	[94, 96]
Hsp90	Direct interaction	Mitochondrial targeting		↑ Ca(2+)-induced opening of the mitochondrial permeability transition pore	[72]
Hsp90	Direct interaction	Protein stabilization		Kinase maturation in epithelial cells	[88]
Receptors and membrane proteins					
β-ENaC	Direct phosphorylation of β-ENaC		Increased protein stability	↑ Salt-scavenging function ↑ Hypertension	[190]
EGFR	Direct phosphorylation of EGFR		Unknown induced effect	↑ EGF receptor signaling	[191, 192]
EGFR	Direct phosphorylation of GRK2	Catalytic activation		↑ Opioid DOR desensitization ↑ Dopamine D3R desensitization	[60, 61]
PDGFR	Direct phosphorylation of GRK2	Catalytic activation		↑ PDGFR phosphorylation and desensitization	[59]
PDGFR	Direct phosphorylation of PDFGR		Ubiquitination and decreased NHERF binding	↓ PDGFR activity ↓ PDFG-induced proliferation and migration in VSMCs	[193]
IGF1R	Co-ipp		Decreased activity	↓ IGF-induced AKT and ERK activation, EGR1 downmodulation in HCC cells	[194]
Cytoplasmic kinases					
p38	Direct phosphorylation of p38		Inhibition	↓ LPS-mediated inflammation	[195]
p38	Unknown		Activation	↑ FcεRI signaling in mast cells	[196]
Akt	Co-ipp		Inhibition	eNOS inhibition in endothelial cells ↑Portal hypertension	[83]
AMPK	Co-ipp and direct AMPK phosphorylation?		Decreased activity	↑ Follicle-stimulating hormone- and AMPK-dependent gluconeogenesis in hepatocytes	[197]
PKC	Direct phosphorylation of GRK2	Catalytic activation and plasma membrane translocation		↑ Phosphorylation and internalization of ErGPCR-2 ↓steroid hormone 20-hydroxyecdysone signaling	[56, 57]
ERK	Direct phosphorylation of GRK2	Catalytic modulation and decreased protein stability		↓ GPCR desensitization ↑ HDAC6 phosphorylation and activation	[65, 69–71]
Src	Direct phosphorylation of GRK2	Catalytic activation and decreased protein stability		↑ GPCR desensitization	[58, 95]
PI3Kγ	Direct interaction		Increased activity	↑ β2AR internalization ↑ AKT and NFAT activation and cardiac hypertrophy	[198, 199]
MEK	Direct interaction		Decreased activity	↓ Chemokine-induced ERK activation	[200]
MST1	Direct phosphorylation of MST1		Increased kinase activity	↑ EGF-induced centrosome separation	[87]
Signaling switchers					
Gαq	Direct interaction		Decreased activity	PLCβ downmodulation ↓cardiac hypertrophy	[63, 79, 201]
RhoA	Direct interaction	Scaffolding of Raf1, MEK1 and ERK2 complexes		↑ EGF-triggered activation of ERK and proliferation in fibroblasts and VSMCs	[202]
Epac1	Direct phosphorylation of Epac1		Decreased membrane targeting	↓ Rap1 activation in neurons	[139]
RalA	Co-ipp		Inhibition	↓ LPA-induced PLC activation in kidney epithelial cells	[203]
Phosducin and phosducin-like protein	Direct phosphorylation of phosducin		Reduced binding to Gβγ	↑ Gβγ-dependent signaling	[204]
eIF3d	Direct interaction	Increased protein stability		↑ AKT/PI3K activity gallbladder cancer cell migration and proliferation	[205]
Signal transducers					
HDAC6	Direct phosphorylation of HDAC6		Increased deacetylation activity	↑ Tubulin dynamics ↑ Pin1-mediated proliferation ↑ Migration/proliferation in tumoral mammary cells and HeLa cells	[71, 111]
Pin1	Direct isomerization of GRK2	Decreased protein stability		G2/M transition	[67]
RKIP	Direct interaction	Decreased kinase activity		↓ GPCR desensitization ↑cardiac contractility “uphold” Raf1 signaling	[206, 207]
eNOS	Co-ipp	Catalytic inhibition by S-nitrosylation		Cardioprotection in ischemia	[73, 208]
APC (adenomatous polyposis coli)	Co-ipp		Increased Auxin/APC complex formation	↓ Wnt1- and Wnt3-induced β-catenin stabilization in kidney epithelial cells and osteoblasts	[209]
Smad1/5/7	Unknown		Increased activity	↑ ALK1 signaling and angiogenesis	[210]
Smad2/3	Direct phosphorylation of Smad2/3		Decreased activity	↓ TGFβ1-induced growth arrest in primary hepatocytes	[211]
IκBα	Direct phosphorylation of IκBα		Degradation	↑ TNFalpha-induced NF-kappaB activation in macrophages	[212]
PDEγ	Direct phosphorylation of PDEγ		Scaffolding of Src and Grb2 complexes	↑ EGF-induced activation of ERK in smooth muscle cells	[213]
IRS1	Phosphorylation and co-ipp		Decreased stability or function	Insulin resistance in different cell types	[158, 214, 215]
GIT	Direct interaction		Scafolding of Rac/PAK/MEK complex	↑ Integrin and S1PR-induced ERK ↑migration (fibroblasts, HeLa cells)	[216]
Cytoskeletal proteins and regulators, protein transport, organelle maintenance					
β-Tubulin	Direct phosphorylation of tubulin		Unknown	Unknown	[217]
α-Actinin	Co-ipp	Catalytic inhibition		↓ GPCR desensitization? GRK2 localization in stress fibers, sarcomere?	[218, 219]
Clathrin heavy chain	Direct interaction	Catalytic activation		↑ Agonist-induced β2AR phosphorylation	[76]
Clathrin heavy chain (CHC)	Direct interaction	Endocytic adaptor for cargo		GPCR internalization	[78]
Clathrin light chain (CLC)	Direct phosphorylation of CLC		Endocytic adaptor for cargo	GPCR internalization	[220]
Ezrin/radixin	Direct phosphorylation of ezrin/radixin		Activation	↑ Epithelial motility in kidney epithelial cells	[221, 222]
α and β-Synuclein	Phosphorylation of synucleins		Reduced binding to phospholipase D2 (PLD2)	Inhibition of synuclein interaction with PLD	[223]
Caveolin 1 and 3	Direct interaction	Catalytic inhibition		↓ GPCR desensitization?	[24]
Caveolin 1	Direct interaction	Protein scaffolding		Isoproterenol-induced eNOS inhibition	[82]
Mitofusin-1 and -2	Direct phosphorylation of Mitofusin-1 and -2		Increased mitochondrial fusion activity?	Mitochondrial resistance to ionizing radiation	[91]
Transcription factors					
Dream	Direct phosphorylation of Dream		Blockade of Kv4.2 membrane trafficking	↓ Peak current density of Kv4.2 channel in kidney epithelial cells	[224]
Period1/2	Phosphorylation of PERIOD1/2		Decreased activity	↓ mPeriod1 transcription ↓PERIOD1/2 nuclear trafficking higher amplitude of PER1 protein rhythms	[117]

A list of selected non-GPCR substrates and interacting proteins of GRK2 is shown. See main text for details

Since the role of GRK2 in different cellular processes and physiological functions is the result of the integration of its canonical and non-canonical interactomes, it is a key to validate these interactions and clearly determine their functional impact in relevant experimental models and to better understand the stimuli and mechanisms linking GRK2 to its potential partners in specific contexts.

## GRK2 structure: a versatile multidomain protein

**Fig. 1:**
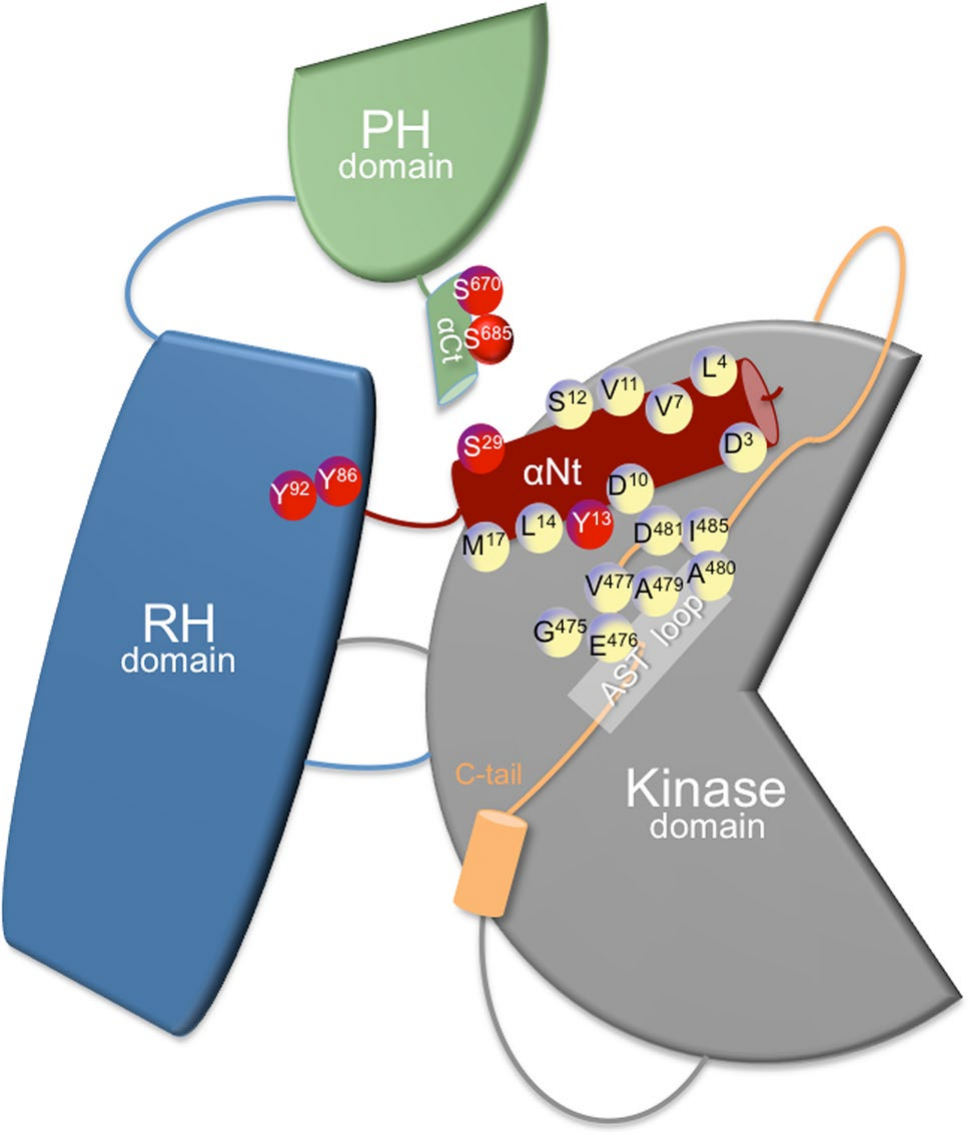
Multi-domain structural organization of GRK2. The three modular domains of GRK2 are sketched out: the N-terminal RGS (regulator of G protein signaling) homology (RH) domain (in blue), the bilobular central kinase domain (gray), and the C-terminal pleckstrin homology (PH) domain (green). The RH domain contacts both the kinase and PH domains and the RH domain–PH domain interface allows a possible route for allosteric communication. Binding of diverse molecules (proteins, phospholipids) to PH and RH domains and post-translational modifications of these domains (Y86, Y92, S670, S685, phosphorylation sites denoted in red) may affect the interface between RH and kinase lobes, and thus GRK2 catalytic activity. Residues indicated in the terminal αN-helix of GRK2 are packed against the AST loop (M17, L14, Y13, D10) and are critical for phosphorylation of GPCRs and cytosolic substrates, highlighting their role in the spatial arrangement of AST and of the kinase domain extension known as C-tail, which is required for kinase domain closure and activity. Other residues of the αN-helix are positioned away from the catalytic hinge and participate in GPCR recruitment, forming a hydrophobic patch for docking (L4, V7, L8, V11, S12) and also conveying allosteric activation to the kinase domain (D3, L4). Some of these exposed residues (D3) are involved in differential interactions with GPCR allowing binding selectivity. Phosphorylation of key αN-helix residues (Y13) or nearby residues (S29) may impact GRK2 catalytic activity and GPCR docking. See main text for details

GRKs are multidomain molecules that emerged at early stages of eukaryotic evolution [22] and that display either ubiquitous (GRK2, 3, 5, and 6), more restricted (GRK4) or cell-type specific (GRK1 and 7) expression patterns [11, 14]. The N-terminal region (αN-helix), characteristic of this protein family and important for receptor recognition, is followed by a domain displaying homology to RGS proteins (thus termed RGS homology, or RH domain), reported to associate with G protein alpha subunits. In the case of GRK2 and GRK3, the RH domain has been shown to interact with GTP-liganded Gαq family members [23, 24]. A central catalytic domain with high similarity to other AGC protein kinases, such as PKA, PKB, and PKC, is shared by all GRKs, whereas a more variable carboxyl-terminal domain is important for the localization and translocation of GRKs to the membrane by means of either post-translational modifications or sites of interaction with lipids or membrane-bound proteins [12, 25] (Fig. 1). Membrane targeting can be achieved via prenylation (in GRK1 and 7), palmitoylation (in GRK4 and 6), a positively charged lipid-binding element (in GRK5) or binding to membrane phospholipids and Gβγ subunits via a pleckstrin homology (PH) domain (in GRK2 and 3).

To date, all mammalian GRKs (except GRK3 and GRK7) have been crystallized (see [26] and references therein). The GRK2 structure in complex with Gβγ subunits [27], followed by the crystallization of GRK2 alone [28] and in complex with Gβγ and Gαq subunits [29], provided exciting insights into GRK regulation. The structural data placed the three defined GRK2 domains (RH, kinase, and PH regions) at the vertices of a triangle and constituted an excellent example of how multiple modular domains can integrate in a single molecule to transduce and modulate signaling events. Such structural design would allow the coordinated control of GRK2 activity and membrane targeting upon GPCR stimulation via dynamic interactions of the different GRK2 domains among themselves and with different intracellular targets [12, 25, 26].

The bilobular fold of the catalytic domain of GRK2 is very similar to that of other AGC kinases and highly conserved among GRKs subfamilies. It consists of an N-terminal small kinase lobe (aa 186–272 and aa 496–513) and a C-terminal large lobe (aa 273–475), sandwiching the catalytic cleft, which is stabilized by the interposition of the αC helix coming from the small lobe. As part of the nucleotide gate, a C-terminal extension to the large lobe, the active site tether (AST, aa 475–485), passes over the cleft contributing to stabilize the nucleotide and phosphor-acceptor binding sites in the active kinase. In many AGC kinases, transitions from inactive conformations (characterized by expanded lobes and disordered nucleotide gate and αC helix) to the active conformation involve phosphorylation of several regulatory motifs at the activation segment/loop, which is located in the large kinase lobe, and at the hydrophobic motif, which is found C-terminal to the small kinase lobe and is sometimes preceded by another important structural determinant, the turn motif. Phosphorylation of these sites coordinates the closure of catalytic lobes and stabilizes the active conformation of the αC helix [30]. In contrast, GRK2 lacks phospho-acceptor sites in the equivalent regulatory motifs, and the RH domain serves as an intra-molecular scaffold that maintains the small lobe of the kinase domain and the αC helix in a competent configuration and helps to maintain the kinase domain in an inactive, open conformation [31–33]. Therefore, activation of GRK2 appears to rely on interaction of its different domains with activating interactors (agonist-occupied GPCR, Gβγ subunits). This allows readjustment of the kinase domain contacts with the N-terminal helix and the RH and PH domains, ultimately leading to allosteric rearrangement of the AST and kinase domain closure (Fig. 1) [12, 25, 26].

Several studies support the notion that multiple GPCR and GRK regions are involved in their interaction [26, 34–36]. The αN-helix appears to stabilize the kinase domain closure via a process that may be regulated by GPCR binding [37–39], and truncation or point mutations of the N terminus of GRKs leads to nearly complete loss of receptor phosphorylation [40–43]. However, it is still not clear to which extent this region is directly involved in receptor binding or in facilitating kinase activity and receptor phosphorylation [26].

Besides the αΝ-helix domain, a proline-rich motif located just before the nucleotide gate has been identified in GRK2 as relevant for receptor binding [44, 45], and several studies suggest an important role for the RH domain of GRKs in receptor interaction [46–50].

In this regard, the recent structure of GRK5 with the β2AR [51] suggests that GRKs display high structural plasticity, showing a dynamic mechanism of complex formation that involves large conformational changes in the GRK5 RH/catalytic domain interface upon receptor binding. These changes facilitate contacts between intracellular loops 2 and 3 and the C terminus of the β2AR with the GRK5 RH bundle subdomain, membrane-binding surface, and kinase catalytic cleft, respectively [51]. Thus, the RH domain would serve as a docking site for GPCRs and help to control kinase activation via transient contacts of the RH bundle and kinase subdomains [26]. Despite all this accumulating knowledge, many questions remain regarding the determinants of GRK preferential targeting toward specific GPCRs, the mechanisms triggering GRK2 kinase activity toward non-GPCR targets, and the control of the scaffolding roles of GRK2 with different signaling partners. As discussed below, the ability of GRK2 domains to interact with activating factors might be modulated by association with additional partners and by protein modifications in response to other signaling networks, thus adapting the functionality of GRK2 to given cellular contexts.

## GRK2 regulation: a precise spatio-temporal control toward the right targets

**Fig. 2:**
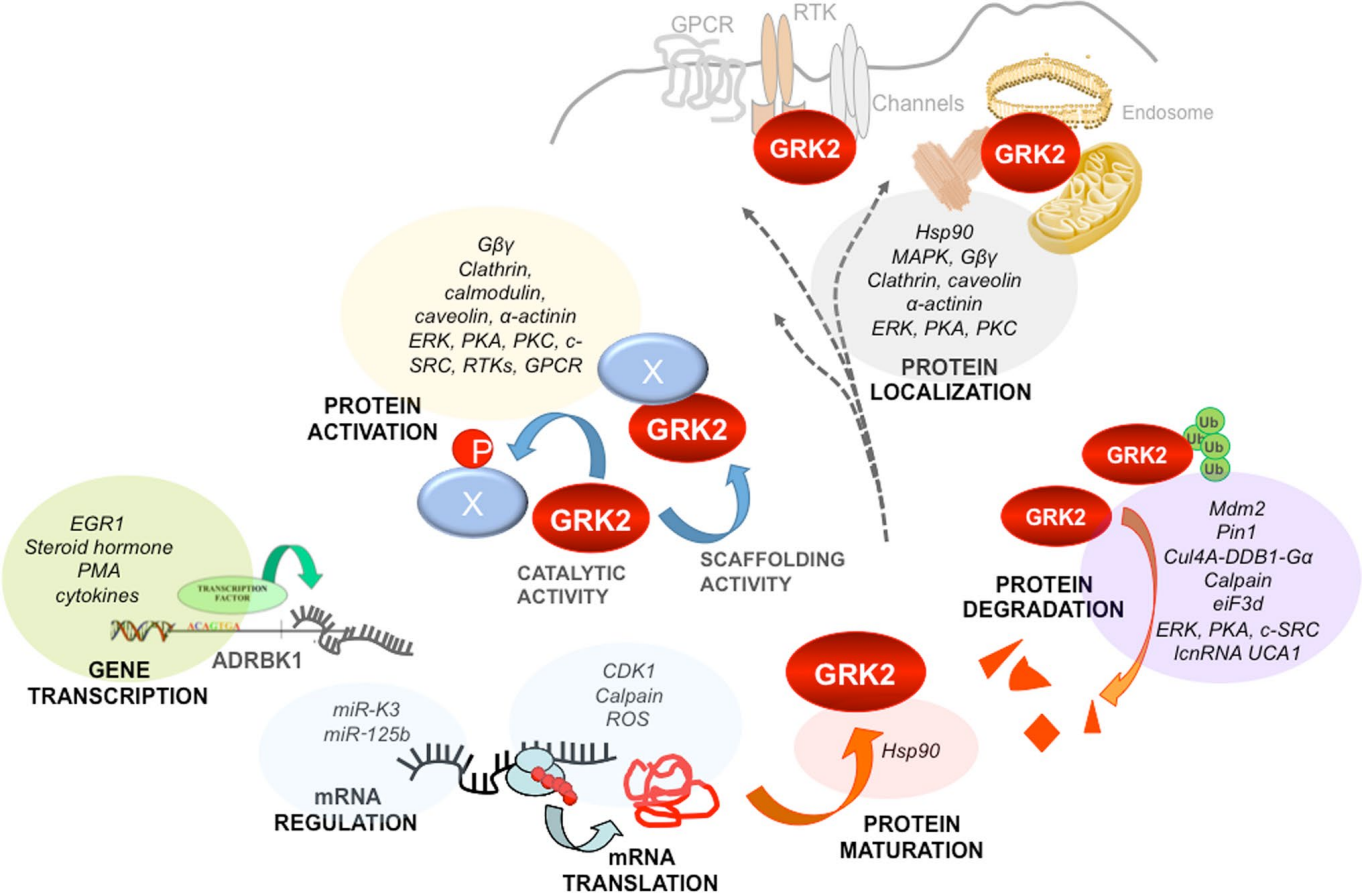
Multiple regulatory mechanisms allow precise spatio-temporal control of GRK2 levels and functionality. Multiple layers of modulation converge to control GRK2 dosage, localization, phosphorylation activity, and scaffolding functions toward both GPCR and non-GPCR partners in response to extracellular signals. Different signals act through transcription factors to modulate transcription of the GRK2 gene (*ADRBK1*). GRK2 transcript levels are finely tuned in diverse settings by miRNAs or via modulation of transcript translation by different mechanisms. Interaction with chaperones (Hsp90) guides GRK2 folding and maturation. A diverse set of cellular inhibitors and activators controls the catalytic activity of GRK2 and its context-specific repertoire of substrates or interactors. Catalytic activity can be influenced by protein–protein interactions in a positive (Gβγ, GPCR) or negative (caveolin, calmodulin, α-actinin) manner. Similarly, phosphorylation by distinct kinases (PKA, PKC, ERK, c-Src, RTKs) or S-nitrosylation also modifies kinase activity and substrate selection. Besides regulating GRK2 activation status, these factors contribute to compartmentalization of GRK2 activity by docking the protein in specific subcellular locations, in close proximity with particular substrates or partners. Finally, GRK2 degradation irreversibly modulates GRK2 activity and levels via ubiquitination by several ligases and proteolytic clearance by the proteasome or calpain proteases. Protein decay is frequently turned on by the same modifications triggering changes in GRK2 catalytic activity, as many phosphorylation sites on GRK2 behave also as enabling signals or phoshodegrons for ligases and proteases. See text for details

Consistent with the complexity of its interactome, GRK2 expression levels, activity, and subcellular location are tightly regulated (Fig. 2). The relative abundance, localization, and activation status of GRK2 in a given cellular context, would directly determine GPCR signaling and desensitization and its interaction with additional partners.

### Post-transcriptional GRK2 modifications shape kinase activity and target selectivity

GRK2 is phosphorylated by other kinases at different residues (Fig. 1), underlying complex cross-talk mechanisms between signaling pathways. GRK2 is modulated by second messenger-directed kinases downstream of many GPCRs. PKA phosphorylates Ser685 at the extreme C terminus of GRK2, enhancing its ability to bind to Gβγ and the activated GPCR, which facilitates homologous desensitization of Gs-coupled GPCRs [52] and also of non-Gs-coupled GPCRs in a heterologous manner via GRK2 membrane targeting. Thus, selective phosphorylation of GRK2 by PKA in response to β-agonists augmented GRK2-Gαq association in response to acetylcholine stimulation of Gq-coupled M3 receptors, leading to inhibition of the Gq effector, PLCβ1 [53]. In turn, PKA phosphorylation of GRK2 is limited by the activity of PDE4 (phosphodiesterase-4), which settles a threshold for GRK2 modification by PKA in several contexts, avoiding inappropriate GRK2 membrane recruitment and receptor desensitization in non-stimulated cells [54, 55]. Phosphorylation by PKC on Ser29 led to an enhanced GRK2 catalytic activity toward GPCRs but not soluble peptides [56]. In the light of structural data pointing that the integrity of the αN-helix of GRK2 (aa 1–30) with preservation of particular acidic residues is key for receptor interaction and phosphorylation [38, 40, 43], it is likely that phosphorylation of Ser29 improves kinase docking to GPCR by shaping properly the intrinsically disordered αN region (Fig. 1). Alternatively, Ser29 modification could exert allosteric effects in GRK2 activation guided by receptor interaction. In addition, phosphorylation of GRK2 by PKC on serine 680 has been reported to translocate the kinase to the cell membrane to desensitize the steroid hormone ecdysone-responsive GPCR (ErGPCR-2) [57].

In addition to PKA and PKC, GPCR stimulation can trigger other kinase cascades to modify GRK2 in a feedback loop, altering its membrane localization and/or catalytic activity. c-Src phosphorylates GRK2 on tyrosine residues located in the αN-helix (Tyr13) and within the RH region (Tyr-86 and Try-92) [13, 58]. Remarkably, the catalytic activity of tyrosine-phosphorylated GRK2 is increased toward both soluble and membrane-bound substrates, suggesting an allosteric effect on the kinase domain. Interestingly, the active site tether (AST) that contributes to stabilization of the nucleotide and phospho-acceptor binding sites in the active kinase makes contacts with the αN-helix. It has been proposed that the side chain of Tyr-13 is packed against the AST residue Val-477, forming a hydrophobic patch required for both receptor docking and allosteric activation (Fig. 1). The Y13A mutant shows reduced phosphorylation of peptides and receptors, which is consistent with a defective stabilization of the active state of GRK2 [40]. It is possible that the gain of a negative charge in Y13 in close proximity to acidic residues (D10, E476) involved in the interaction with the AST loop might alter receptor docking specificity and substrate preferences. Residues Y86 and Y92 lie proximal to a hydrophobic interface formed between the RH domain and the kinase large lobe (Fig. 1), and their phosphorylation could influence kinase domain closure, although this remains to be proved. Interestingly, activation of GRK2 by means of tyrosine phosphorylation can be achieved independently of GPCR stimulation and β-arrestin-mediated recruitment of c-Src [13]. PDGFRβ itself phosphorylates and activates GRK2, which then serine-phosphorylates and desensitizes PDGFRβ in a negative feedback loop [59]. EGFR also directly phosphorylates GRK2 on the reported tyrosine residues, allowing membrane recruitment and enhanced GRK2-mediated desensitization of opioid receptors [60] or dopamine D3 receptors [61] in a EGF-dependent manner. Even membrane receptors devoid of catalytic activity can also engage cytosolic tyrosine kinases to activate GRK2. TCR-activated c-Src leads to inducible tyrosine phosphorylation of GRK2 and stimulation of GRK2-dependent CXCR4-Ser-339 phosphorylation and TCR-CXCR4 complex formation. Such GRK2-mediated complex drives the PI3Kγ-dependent recruitment of PREX1, which is required for full cytokine secretion during T cell activation [62].

In addition to increasing GRK2 activity, tyrosine phosphorylation within the RH domain also enhances the interactions of GRK2 with other proteins such as Gαq upon muscarinic receptor M1 stimulation [63] or the scaffold protein GIT1 in response to integrin receptor activation ([64], see also below). Thus, tyrosine phosphorylation of GRK2 is a key event in receptor transactivation that endows this kinase with novel catalytic and non-catalytic effector capabilities.

Different proline-directed kinases, which play critical roles in cell cycle, stress responses, and survival or metabolic control, converge in phosphorylating GRK2 on Ser-670. Mitogen-activated protein kinases ERK1/2 and p38 have been reported to phosphorylate this residue in response to GPCR [65] or TLR4 [66] activation, and cyclin-dependent kinase, CDK2–cyclin A, phosphorylates GRK2 during cell cycle progression [67]. In a setting of ischemia, PI3K/AKT pathway activation promotes S670 phosphorylation through an unidentified kinase [68]. The S670 residue lies within the Gβγ-binding domain of GRK2 (Fig. 1), and its phosphorylation strongly impairs the interaction of GRK2 with Gβγ subunits, thereby inhibiting kinase translocation to the plasma membrane and kinase activity toward membrane-located substrates [69, 70]. Phosphorylation of GRK2 on S670 might alter potential paths of GRK2 allosteric activation. The RH domain has been proposed to act as an allosteric transducer domain, since the interaction of the α10 helix (aa 511–515) with the small kinase lobe may play regulatory roles similar to the SH2-catalytic domain linker in c-Src [27]. Close to this RH–kinase small lobe interface, the terminal subdomain of the RH region interacts with a cluster of residues in the PH domain that binds directly to Gβγ. This suggests that S670 phosphorylation may transmit structural alterations affecting the conformation of the catalytic domain and thus activity. Interestingly, this modification at the C terminus promotes a switch in GRK2 substrate specificity, fostering the acquisition of a distinctive competence to phosphorylate HDAC6 [71], also see below). In addition, GRK2 phosphorylation at S670 by MAPK disrupts GRK2 interaction with GIT1 [64], whereas it is required for kinase localization to the mitochondrial outer membrane via enhanced interaction with the chaperone Hsp90 [72]. In summary, S670 phosphorylation appears to play a central role in the modulation of GRK2 functional features such as catalytic activity toward GPCRs and non-GPCR substrates, subcellular localization, and the ability to interact with protein partners (see Fig. 2).

Regarding other types of post-translational modifications, GRK2 can undergo S-nitrosylation of Cys residue 340 within its catalytic domain, leading to the inhibition of kinase activity toward GPCRs [73], as well as ubiquitination of lysine residues related to the control of GRK2 turnover (see below).

### GRK2 is present in different subcellular pools

GRK2 was initially identified as a cytosolic protein able to translocate to the plasma membrane upon GPCR stimulation [4, 13]. However, a significant amount of GRK2 was reported to be associated with internal microsomal membranes by means of electrostatic interactions between the N-terminal region of the kinase (RH domain) and an unknown protein which is an integral component of the microsomal membrane [74, 75]. Interestingly, the interaction of GRK2 with the unidentified anchoring protein leads to inactivation of the bound kinase, what can be reverted by stimulation of endogenous heterotrimeric G proteins [75]. On the other hand, the C terminus of GRK2 directly binds to clathrin, an interaction that may facilitate the internalization of certain GPCRs, resulting in the co-localization of GRK2 with β-adrenergic receptors in endosomes during agonist-induced receptor internalization [76–78].

GRK2 activity is inhibited upon interaction with several cellular partners such as calcium-binding proteins, α-actinin, Hsp90, and caveolin (reviewed in [79]). For instance, calmodulin interacts with GRK2 at sites located at both the N- and C-terminal domains of the kinase and directly inhibits its activity [80]. Of note, these regulatory partners may act as adapters or anchoring factors, helping to preserve different pools of inactive kinase and to guide catalytic or scaffolding activities of GRK2 toward particular substrates (Fig. 2). In this regard, the association of the kinase with caveolin-1 or -3 inhibits GRK2-mediated phosphorylation, suggesting that caveolins may play a role in controlling basal GRK2 activity [81]. However, since caveolins serve as scaffolds for a variety of signaling molecules including GPCRs, different MAPKs, and G proteins helping to compartmentalize their signaling, the association of the GRK2 with caveolin may also facilitate the interaction of this kinase with other signaling or regulatory molecules. In this regard, caveolin and GRK2 together regulate eNOS in sinusoidal endothelial cells. β2AR stimulation triggers the association of both proteins, and β-agonist-induced Tyr-14 phosphorylation of caveolin is required for such association. Interestingly, the GRK2–caveolin complex leads to eNOS recruitment and decrease of AKT-mediated eNOS-Ser1177 phosphorylation, resulting in blockade of NO production [82]. Of note, GRK2 has been reported to interact directly with AKT in liver sinusoids, thus inhibiting AKT-mediated phosphorylation of substrates [83], but whether such regulatory effect is mediated by caveolin has not been addressed.

Mitochondrial and nuclear pools of GRK2 have also been reported. Although GRK2 does not display a canonical nuclear localization sequence as GRK5 does [84], high levels of both GRK isoforms have been detected in the nuclear fraction of neuronal cells from the striatum [85]. Interestingly, like GRK5 [86], GRK2 has been found in centrosomes and was suggested to play a critical role in mediating EGF-dependent separation of duplicated centrosomes, although it has no role in centrosome duplication or microtubule nucleation [87].

GRK2 has also been shown to interact with heat shock protein 90 (Hsp90), a known mitochondrial chaperone [88], to localize in the mitochondria and to regulate mitochondrial biogenesis when accumulated inside this organelle [89]. GRK2 mitochondrial levels increase during inflammation or upon LPS stimulation in macrophages, helping to facilitate biogenesis and to restore mitochondrial function [90]. It has also been proposed that GRK2 localizes to mitochondria in HEK293 cells upon ionizing radiation and that that this kinase plays a protective role in this context by regulating mitochondrial fusion through interaction and phosphorylation of Mitofusin-1 and -2 [91]. On the contrary, in the context of ischemia reperfusion in vivo and in cultured myocytes, GRK2 localizes to mitochondria via ERK-mediated phosphorylation of GRK2 at Ser670 and Hsp90 binding, and contributes to mitochondrial-dependent pro-death signaling [72], increases superoxide levels, and impairs fatty acid oxidation and ATP production [92]. A better understanding of the stimuli triggering GRK2 localization to mitochondria and the impact of GRK2 dosage on mitochondrial function in different cell types and pathophysiological contexts is needed.

### Control of GRK2 protein turnover

Protein degradation has emerged as the main mechanism for triggering rapid local changes of GRK2 functionality (Fig. 2). GRK2 is a short-lived protein and the chaperone function of Hsp90 plays a critical role in GRK2 protein stability and maturation in most cell types. Impaired Hsp90 activity results in degradation of GRK2 in a proteasome-mediated manner [88]. Moreover, GRK2 is rapidly degraded upon GPCR activation in an ubiquitin and proteasome-dependent manner [93]. Ubiquitination of GRK2 at the N-terminal lysine residues K19, K20, K30, and K31 marks the protein for its proteasome-dependent degradation in basal and agonist-stimulated conditions [94]. Interestingly, both GRK2-mediated phosphorylation of ligand-occupied receptors and β-arrestin engagement of the receptor complex are early common events triggered by GPCR activation, and both were found to be necessary for agonist-induced proteolysis of GRK2. Further studies revealed that cSrc-dependent phosphorylation of GRK2 was instrumental in this process [95]. Moreover, ERK-mediated phosphorylation of GRK2 at Ser670 also can promote its degradation [65]. Although both c-Src- and ERK-mediated phosphorylation of GRK2 can target GRK2 for degradation independently, ERK preferentially phosphorylates once GRK2 has been previously phosphorylated on tyrosine residues by c-Src [65]. The main E3 ligase implicated in GRK2 turnover by the proteasome pathway is Mdm2 [94]. Further characterization of the Mdm2-dependent GRK2 degradation revealed that upon GPCR activation, Mdm2 action on GRK2 is mediated by previous GRK2 phosphorylation on Ser670 but not by tyrosine phosphorylation, although c-Src phosphorylation facilitates the subsequent phosphorylation of GRK2 by MAPK [96].

Arrestins are known to recruit c-Src, ERK, and Mdm2 to the vicinity of activated GPCRs [97, 98] and these scaffold molecules are required to facilitate sequential GRK2 phosphorylation and Mdm2-mediated degradation upon GPCR activation. However, in the absence of GPCR activity, β-arrestins do not participate in the Mdm2-mediated regulation of basal GRK2 turnover, but instead compete with GRK2 for Mdm2 and suppress basal GRK2 degradation [96]. In basal conditions, c-Src is able to mediate Mmd2-independent GRK2 degradation, although the E3 ligase implicated in this process remains to be elucidated. Thus, β-arrestins play a coordinating role recruiting kinases and/or ubiquitin ligases to GRK2 in the basal condition or upon activation of GPCRs, regulating GRK2 turnover via different pathways. Adding more layers of complexity, it is also worth noting that Mdm2 phosphorylation by AKT in response to stimulation of growth factor receptors such as IGFR leads to E3 ligase translocation to the nucleus, resulting in enhanced stability of cytoplasmic GRK2 and higher expression levels [94].

Notably, the phosphorylation status also affects GRK2 stability in a cell cycle context, thus governing the fluctuations in kinase levels taking place during cell cycle progression. Phosphorylation of GRK2 at Ser670 by CDK2–cyclin A and the subsequent binding of the prolyl-isomerase Pin1, are required for transient GRK2 proteasome degradation during the G2/M transition [67], although the E3 ligase involved was not identified. As Mdm2 protein expression is also cell cycle regulated, with a prominent accumulation through G2/M, it is tempting to suggest that this ligase plays a role in downregulating GRK2 in this context, but the contribution of other ligases cannot be excluded.

It has been reported that Gβ subunits have a non-canonical function acting as substrate recruiters for Cul4-RING ubiquitin ligases [99, 100]. In particular, a member of the Gβ family, Gβ2, specifically targeted GRK2 for ubiquitination and degradation by the cytosol-enriched DDB1-Cul4A-ROC1 ligase. Curiously, agonist stimulation of β2AR caused delayed turnover of GRK2 and up-regulation of global kinase levels in HEK293 cells through a mechanism involving PKA-mediated phosphorylation of DDB1 and disruption of Gβ2 binding to DDB1-Cul4A ligase. This mechanism of feedback regulation and GRK2 stabilization evoked by β2AR stimulation appears to be in conflict with previously published data demonstrating GPCR-(including β2AR)-induced GRK2 degradation [101]. Moreover, while these authors suggest that DDB1-Cul4A-ROC1 ligase is the physiological ligase impaired in cardiac hypertrophy, heart failure or hypertension, conditions linked to adrenergic overdrive and excessive PKA activation, others have demonstrated that defective cardiac Mdm2 led to a significant increase in GRK2, resulting in severely impaired cardiac function [102]. Therefore, further studies are needed to define the main E3 ligase responsible of keeping steady-state levels of GRK2 in defined pathological contexts. Interestingly, Cul4-DDB1 is also recruited to the non-classical GPCR Smo through Gβ to promote internalization and degradation of Smo receptor and GRK2 itself, while in the presence of Hedgehog, PKA is activated and Cul4-DDB1 is dissociated from Smo, allowing receptor and GRK2 accumulation for signal transduction [99]. Based on this finding, it has been suggested that this ubiquitin ligase could play a broader role in GPCR regulation, being recruited as active receptors by receptor-bound GRK2-Gβ complexes to trigger their internalization, in a similar way as GPCR-bound β-arrestins bring Mdm2 and other ubiquitin ligases for GRK2 and receptor ubiquitination.

On top of that, other protein degradation machineries can participate in the control of GRK2 levels. Inappropriate calpain activation has been implicated in various disease states including cerebral and heart ischemia, muscular dystrophies, and chronic inflammation [103]. Proteasome-independent GRK2 downregulation takes place in several of these settings. In arthritis and in active relapsing–remitting multiple sclerosis (MS) or secondary progressive MS [104], inflammatory cytokines trigger oxidative stress-induced GRK2 down-regulation through the calpain proteolytic pathway. Many substrates of calpains exhibit a PEST region, rich in proline (P), glutamic acid (E), serine (S), and threonine (T). GRK2 bears a putative PEST region (aa 591–615) and purified calpain is capable to promote partial proteolysis of recombinant GRK2 in a calcium-dependent manner in vitro, suggesting that the cleavage may occur between aa 677 and 678 of GRK2 [105]. Of note, the existence of a cAMP-dependent protein kinase A (PKA) phosphorylation site (S685) in GRK2 close to the calpain cleavage site raises the possibility of a regulatory interplay between protein phosphorylation and calpain-mediated degradation of GRK2 in the context of myocardial and cerebral ischemia, which are conditions characterized by a potent activation of PKA.

### GRK2 regulation at the transcriptional level

Although regulation of protein stability appears to play a major role in determining GRK2 dosage, changes in mRNA levels of GRK2 have been noted in several pathological conditions, including hypertension, heart hypertrophy, and heart failure [18, 106], in the acute stage of infectious diseases, such as pneumonia and sepsis [107], or in tumoral pathologies [19]. However, relatively little is known regarding the mechanisms controlling GRK2 promoter activity and transcript stability or other layers of control, such as regulation by non-coding RNAs or RNA-binding proteins (Fig. 2).

Several SNPs within the promoter of the *ADRBK1* gene and in non-coding regions have been identified. The rs4930416 and rs1894111 SNPs were found to be associated with differential antihypertensive drug response in black and white patients [108]. Several of these SNPs within the gene were found to be in linkage disequilibrium (LD) in the black population, what could indicate that some of these SNPs may impact gene regulatory sequences. Although the influence of non-coding SNPs in GRK2 expression has not been addressed, in silico analysis showed that they may yield unique transcriptional factor-binding sites, resulting in potential changes in *ADRBK1* regulation [109].

Analysis of the human breast cancer TCGA database shows that GRK2 mRNA is expressed at statistically significantly higher levels in tumor cells compared to normal breast tissue [110], which is in line with increased protein levels detected in breast cancer patients [111]. Interestingly, the *ADRBK1* gene is located at the 11q13.2 band and is identified as part of the amplicon 11q13 which undergoes amplification at high frequencies in breast tumors, showing a strong association between copy number status and gene expression level [112].

In vascular cells, phorbol esters (PMA), as well as the activation of Gαq or α1-adrenergic signaling pathways, increased GRK2 promoter activity, consistent with increased GRK2 protein in settings of physiological vasoconstriction and hypertrophy, whereas pro-inflammatory cytokines had the opposite effect [13, 113]. Mitogenic stimulation led to increased GRK2 mRNA expression in T cells [114] and increased GRK2 protein levels in different cell types [111, 115]. However, the identity of transcription factors downstream of such stimuli that are responsible for GRK2 mRNA alterations is largely unknown. It has been reported that several regions of the GRK2 promoter match with the response element of EGR-1, a transcription factor stimulated by PMA, and usually engaged in responses stimulated via Gαq-coupled GPCRs. EGR-1 binds to GRK2 promoter and a novel G(-43)A polymorphism (rs182084609) was identified within one of these EGR-1 binding sites regions, leading to a greater increase in EGR-1-induced transcriptional activity in the promoter bearing the G(-43) allele vs. the A(-43) allele [116].

Emergent findings point to a potential reciprocal interplay between GRK2 and the circadian rhythms machinery, based on the effect of GRK2 on the Period 1 and 2 (PER1/2) negative clock regulators [117, 118]. GRK2 acts post-translationally on PER1 and PER2, physically binding to both proteins and promoting the phosphorylation of PER2, what impairs the trafficking of PER proteins into the nucleus and causes sustained Per2 expression, disrupting circadian rhythms. Interestingly, the mRNA levels of GRK2 in different tissues (such as colon and liver) appear to oscillate in a circadian manner as shown in the CircaDB database (http://circadb.hogeneschlab.org/query), suggesting that GRK2 may be a clock-controlled gene.

MicroRNAs (miRNAs) are short non-coding RNAs (ncRNAs) that are emerging as important modulators of the expression of multiple genes via degradation of mRNA or by preventing translation of target genes. Increasing evidence demonstrates that long non-coding RNAs (lncRNAs) also regulate gene and protein expression via transcriptional and post-transcriptional processes [119, 120]. However, little is known about the participation of these factors in GRK2 regulation. It has been described that miR-K3, a Kaposi’s sarcoma-associated herpesvirus (KSHV) miRNA, facilitates cell migration and invasion via activation of the CXCR2/AKT pathway by repressing GRK2 expression [121], pointing to a role of GRK2 in suppressing KSHV-associated tumor progression. Besides migration and invasion, miR-K3 also enhances KSHV latency and angiogenesis by targeting GRK2 and inhibiting its expression [122]. The lncRNA UCA1 increases the ability of gastric cancer cells to metastasize by regulating the stability of GRK2 protein via the promotion of Cbl-c-mediated ubiquitination and degradation of GRK2, adding another level of complexity to the regulation of the expression of this kinase [123]. The identification of miRNAs and lncRNA that are able to control GRK2 dosage in different conditions is an interesting future research avenue.

## Revisiting paradigms on GPCR-mediated GRK2 activation

The conventional flowchart in GPCR homologous desensitization starts with agonist receptor occupancy and coupling of the activated receptor with heterotrimeric G proteins which dissociate into Gα subunits and Gβγ dimers for downstream signal transduction. Released Gβγ subunits and plasma membrane phospholipids co-recruit cytoplasmic inactive GRK2 into the vicinity of ligand-bound GPCR, and they together allosterically activate the kinase for receptor phosphorylation, followed by high affinity association of β-arrestins with the phosphorylated receptor [2, 4]. Therefore, this paradigm implies that: (1) GRK2 exclusively phosphorylates GPCR in their agonist-bound state; (2) G protein activation and hence GRK2-Gβγ complex formation is mandatory for receptor phosphorylation; (3) β-arrestin engagement only occurs upon GRK2-mediated GPCR phosphorylation. However, recent functional and structural data have called into question these principles. For instance, GRK2 can be docked directly to some GPCRs in a Gβγ-independent manner [124]. By using altered dopamine D2R receptors, prone to preferentially stimulate G proteins D2R[Gpro] or recruit β-arrestins D2R[βarr], in combination with biased ligands directing wild-type D2R toward β-arrrestin in a GRK2 dosage-dependent manner, these authors demonstrated that G protein-independent recruitment of GRK2 is key for preferential D2R-biased signaling to β-arrrestin. GRK2 bound to D2R in such conditions is active toward the receptor and its phosphorylation promotes β-arrestin mobilization. The fact that GRK2 can be recruited to wild-type D2R receptors with a rather modest contribution from Gβγ-mediated kinase translocation poses the question of how kinase activation occurs in the presence of different receptor conformations, and whether the catalytic competence of GRK2 in such GPCR-only complex is the same than in the presence of Gβγ subunits (for instance, the extent and identity of phosphorylated residues in D2R[βarr] and D2R[Gpro] could be different).

Other studies support the notion that GRK2 docking to GPCRs and activation can be uncoupled from Gβγ-mediated recruitment. Data obtained with the catalytically inactive GRK2-K220R mutant suggested that kinase activity was dispensable for plasma membrane and GPCR recruitment since this mutant is able to interact with Gβγ subunits. Thus, reduced efficacy of β-arrestin recruitment in the presence of the GRK2-K220R mutant was ascribed to the lack of GPCR phosphorylation by a receptor-bound inactive GRK2. However, pharmacological inhibition of GRK2 with the small molecule Cpmd101 yields contradictory results when compared to GRK2-K220R in receptor recruitment assays. Neither dopamine D2R interaction with Cmpd101-bound GRK2 nor with K220R mutant protein is affected [124], while the interaction of GRK2 with MOR opioid receptor is fully prevented by Cmpd101 [125]. It seems that for some GPCRs, GRK2 must be in a pro-active configuration for interaction and already folded in the context of a K220R mutation, which only impedes phosphotransferase activity. In contrast, the inactive conformation of GRK2 stabilized by Cpmd101, that impedes the closure of kinase domain [126], is competent to interact with other GPCRs, suggesting that concurrent events can contribute differently in the recruitment of active vs. non-active GRK2 (Gβγ-preassembled complexes, interacting partners, post-transcriptional modifications). This diversity in GRK2 receptor docking might have implications, for instance, in whether Cpmd101 can prevent scaffolding roles of GRK2 in the desensitization of Gαq-coupled receptors.

Overall, these results suggest that GRK2 (in either active or inactive forms) could interact with GPCRs in different conformational states, and that different paths of allosteric activation could trigger GRK2 catalytic activity.

As discussed in the previous sections, the control of GRK2 activation involves dynamic interactions of its different domains and regions with GPCR, Gβγ, and lipids and among themselves, ultimately leading to rearrangement of the AST loop and kinase domain closure, and this activation process can also be influenced by post-translational modifications in the N-terminal RH domain and the C-terminal PH domain of the kinase (Fig. 1).

In addition to AST, the first 20 amino acids of GRKs are also quite disordered and, based on recent crystallographic studies on GRK6, it has been proposed that this region may adopt an extended α-helix conformation that, in addition to serving as an interface with GPCR, would pack against the kinase small lobe and the AST, contributing to the stabilization of active conformation, and induce allosteric activation. Thus, activated GPCRs could stimulate GRKs by ordering the N terminus and AST regions. Furthermore, the N-terminal region of GRKs could play a role in kinase domain closure, even independently of receptors [41].

Remarkably, both AST and the αN-helix regions differ broadly in their sequences among different GRKs, suggesting that they might convey diverse ways of allosteric activation. The identification of key residues for receptor docking and/or kinase allosteric activation in the N-termini of GRKs may help to understand GPCR phosphorylation preferences by GRKs or how GRK2 might be allosterically activated by non-target GPCRs to trigger phosphorylation of additional substrates. Mutational analysis combined with BRET-based protein interaction and kinase phosphorylation assays has allowed the identification of key surface-exposed residues (L4, V7, L8, V11, S12) in the αN-terminal helix of GRK2 that comprises a direct hydrophobic GPCR docking site able to provide GPCR selectivity (Fig. 1). For instance, mutant GRK2-L4A is impaired in α2AR but not β2AR interaction, while other mutations strongly increased kinase recruitment to α2AR [38, 40]. Differential impact of αN residues in GRK2-mediated phosphorylation of GPCRs and soluble peptides also indicates that some positions facing either the small lobe (A16, M17) or the AST loop (D10, Y13) (see Fig. 1), contribute to induce active conformations with different catalytic outcomes. Receptor docking sites on the αN (such as D3 or L4) also relay allosteric activation, as receptor-induced phosphorylation of soluble peptides is impaired when theses residues are mutated. Interestingly, these meticulous mutational studies also suggest that receptor kinase docking can be uncoupled from receptor-induced kinase allosteric activation. This is exemplified by the mutant GRK2-D10A which showed compromised catalytic activity toward soluble peptides and several GPCRs, despite its efficient docking, pointing to a poor active conformation [38, 40]. Intriguingly, this mutant fulfills phosphorylation of tubulin, while other αN mutants are competent to phosphorylate receptors but not soluble peptides. These findings suggest that flexibility at the interface of αN residues and the AST loop may confer biased kinase activity. Phosphorylation of GRK2 or interacting partners affecting the αN region of GRK2 may not only quantitatively regulate the catalytic activity (as phosphorylation by PKC on S29 in the context of GPCR or peptide phosphorylation) but could qualitatively enable catalytically active conformations toward specific targets. This scenario becomes more plausible as the list of non-GPCR and non-membrane substrates of GRK2 grows in number and heterogeneity (see Table 1), raising questions about how closure of kinase domain is achieved in a receptor- and Gβγ/phospholipid-independent manner.

## Examples of context-specific GRK2 multifunctionality

### Multifunctional roles of GRK2 in the modulation of pain perception and chronicity

Pain hyper-sensitivity and chronic pain are disabling conditions conditioned by genetic and environmental factors. In the acute phase of pain, inflammatory mediators released by damaged tissues and catecholamines secreted by sympathetic afferents and adrenal gland under stress provoke sensitization of nociceptors to noxious stimuli in a process known as hyperalgesia, which in the short term is beneficial for the organism. Hyperalgesic factors directly stimulate GPCRs in nociceptor cells such PGE2 receptor (EP2) or β2AR among others, which activate cAMP and PKA-dependent pathways that reduce hyperpolarisation-activated inward currents and augment voltage-activated Na currents, thus increasing the frequency of action potential firing [127]. Although inflammatory stimuli or adrenergic stress typically result in short-lasting hypersensitivity, they can also evoke a priming effect that triggers more pronounced and persistent sensitization to subsequent hyperalgesic challenges or to noxious stimuli, thus contributing to chronic pain.

In acute hyperalgesia, GPCR desensitization curtails cAMP and PKA activation by either conventional GRK2-dependent receptor phosphorylation (β2AR) or by undefined GRK2-independent mechanisms (EP2) [128]. Notably, even though long-term exposure to PGE2 causes desensitization of receptor-mediated cAMP production and activation of PKA, PGE2-induced pain sensitization is not downregulated, i.e., successive challenges result in stronger and more prolonged pain responses [129]. Interestingly, inflammation produces a decrease in GRK2 levels in nociceptors, and reduction of GRK2 levels in peripheral sensory neurons by a variety of approaches enhanced intensity and duration of epinephrine- or PGE2-induced mechanical hyperalgesia [130–134]. Conversely, delivery of extra GRK2 prevented the prolongation of PGE2-induced hyperalgesia in primed mice [135]. These data strongly support that GRK2 is key in the control of pain priming and chronicity.

Transient hyperalgesia is a PKA-dependent process, while persistent pain is PKA independent and involves PKCε/ERK-mediated pathways that ultimately phosphorylate transducer proteins and ion channels in peripheral sensory terminals, leading to their enhanced activation [136]. PKCε is a key intracellular mediator implicated in the onset of mechanical, thermal, or inflammatory hyperalgesia, and in transition from acute to chronic inflammatory pain. In naïve animals, acute PGE2 and epinephrine activate the cAMP-PKA pathway [131, 132, 137], while in primed animals and cellular models, agonist-induced cAMP increase results in activation and membrane translocation of Epac1, which stimulates PKCε via phospholipases, PLC and PLD [138]. Importantly, low levels of GRK2 in nociceptors switch EP2- and β2AR-induced signaling from a protein kinase A-dependent to a PKCε-dependent pathway [134]. GRK2 basally interacts with Epac1 and GRK2-mediated phosphorylation of Epac1 on S108 precludes Rap1 and PKCε activation, without apparently affecting the cAMP binding to Epac1 or cAMP-Epac1 activation of Rap1 [139]. Thus, GRK2 restrains the ability of Epac1 to stimulate local targets by means of altering the regulatory domain that mediates the subcellular localization of Epac through binding to phosphatidic acid in the plasma membrane.

Opposed to the role of inflammatory or adrenergic receptors in hyperalgesia, stimulation of opioid receptors in the cell bodies in the dorsal root ganglion (DRG) and on primary afferent neurons triggers analgesia. Opioids act on different types of GPCRs (MOR, DOR, and KOR) coupled to Gi/o proteins that reduce the cAMP levels and PKA activity, thus causing nociceptor hyperpolarization and decreased neuronal excitability. Interestingly, GRK2 can trigger rapid phosphorylation and desensitization of MOR [125], promote downregulation of KOR in diabetic conditions [140] and in response to specific opioid ligands [141] or enable internalization and recycling of DOR [142]. A recent report has also indicated that phosphorylation of GRK2 at S670 is higher in female that in male mice in both ventral striatum and in spinal cord, leading to decreased KOR-mediated G protein signaling to ERK and defective agonist-induced analgesia in female animals, a gender difference that can be rescued by inhibition of GRK2 catalytic activity in females [143]. Interestingly, chronic pain is more common in women. Nociception itself is influenced by sex due to differential cortical processing of pain-related stimuli [144], whereas the sensitivity of peripheral sensory neurons is apparently also under the influence of estrogens [145], by mechanisms involving rapid non-genomic signaling in nociceptors via the plasma membrane estrogen-sensitive GPCR GPR30 (potential target of GRK2) and palmitoylated-estrogen receptors, ERα and ERβ [146]. These results open the possibility that gender-specific differences in GRK2 expression and/or phosphorylation status might underlie gender differences in pain perception.

In summary, both GPCR- and non-GPCR-related functions of GRK2 play a role in the control of pain. It would be of interest to determine whether changes in GRK2 dosage taking place in inflammatory pain conditions differentially affect the role of this kinase in the modulation of the EPAC1 pathway and in the control of both hyperalgesic and analgesic GPCR signaling.

### Catalytic and scaffold roles of GRK2 modulate proliferation and cell cycle progression

The driving role in the coordination of cell growth and division has been traditionally ascribed to tyrosine kinase growth factor receptors [147]. However, it is now undisputed that GPCRs also are relevant players in the regulation and activation of cellular growth pathways [148]. Many potent mitogens such as lysophosphatidic acid (LPA), thrombin or prostaglandins stimulate cell proliferation through GPCR activation [148, 149]. The complex interactome of GRK2 and its ability to regulate both GPCRs and RTKs responding to a vast diversity of external cues (nutrients, stress and metabolic hormones, growth factors) make this protein a potential signaling integrator linking cell proliferation to environmental constrains. The varied mechanisms underlying the role of GRK2 in GPCR and RTK-triggered MAPK cascades have been recently reviewed [12].

In addition to tuning mitogenic, growth arresting, and survival signaling pathways, emerging evidence indicates that GRK2 also modulates the cell cycle and division machinery. GRK2 appears to contribute to proper and timely progression of G1/S and G2/M transitions of the cell cycle by targeting specific components of the cell division machinery [67]. Reduction of BrdU-positive cells in GRK2 knockdown zebrafish embryos and in model cell lines [150] point to a role of GRK2 in G1/S. During G1, cells synthesize mRNA and proteins for mass and volume increment before entering into the S phase. This process is controlled and limited by growth factors, the extracellular matrix, and cell–cell contacts, as well as by stress conditions [151]. Also, cell division is not allowed until a defined cell homeostatic size is achieved; otherwise dividing cells will become smaller. Cells can assess size thresholds based on protein synthesis rates, which are influenced by nutrient and growth factor signaling [152]. Cyclins and other CDK activators such as Cdc25 phosphatase might link signaling routes of sizing with cell cycle transitions at G1/S and G2/M. In this context, the influence of GRK2 in nutrient sensing and/or the intensity of growth signaling pathways might be key for cell division. For increasing cell size, ribosomes and the translational machinery need to accumulate, and phosphorylation of the ribosomal S6 protein by S6 kinase (S6 K) is important in this process. S6 K is regulated directly by mTORC1, which is a master sensor of nutrients and a translational activator of cell cycle initiators such as cyclin D [153]. Interestingly, positive correlations between mTOR activity and GRK2 protein levels have been reported in different cellular models [154, 155], although whether GRK2 is an upstream modulator of mTORC1 or a downstream effector is not known. It has also been suggested that the mTORC1 pathway is modulated by GPCRs through REDD1 (regulated in development and DNA damage responses), a stress-responsive factor that binds to and inhibits mTORC1. Diverse GPCRs known to be regulated by GRK2 recruit REDD1 to the plasma membrane, thus relieving mTORC1 inhibition [156]. Therefore, GRK2 may influence (either at the receptor or post-receptor level) cell growth during the gap phases of the cell cycle via mTORC1 pathway modulation. On the other hand, GRK2 phosphorylates the ribosomal component P2 in a β2AR-dependent way, fostering the translational activity of ribosome 60S subunits [157], and is known to bind and phosphorylate IRS1, other relevant modulator of cellular proliferation [18, 158], thus providing other potential avenues for GRK2-mediated control of cell proliferation during the G1 phase.

The complex mechanisms leading to transient GRK2 protein downregulation during G2/M (as detailed in a previous section), together with the delayed progression in this phase when GRK2 is improperly stabilized [67] and its localization at the centrioles and pericentriolar matrix in both interphase and during centrosome separation [87], suggest that GRK2 participates in processes that help to prepare the cells for entry into the mitosis phase [159]. During G2, the so-called “centrosome disjunction” process is mediated by the NIMA-related kinase Nek2, which phosphorylates C-Nap1 and rootelin to allow centrosomes to separate in a kinesin (Eg5)-mediated manner and form the two poles of the mitotic spindle [160]. Such centrosome separation must be precise, and its improper timing or length may result in defective mitotic spindles [161, 162]. Of note, GRK2 is involved in the timely regulation of this process by directly phosphorylating Mst2 kinase (an upstream activator of Nek2) in response to EGF receptor stimulation during the early course of G2 [87], thus configuring a GRK2/Mst2/Nek2 kinase cascade. EGFR phosphorylation of tyrosine residues in GRK2 may foster its catalytic activity toward Mst2, which could add to the disjunction mechanism triggered by PLK1 in prophase to make centrosome separation faster and earlier in G2.

Downregulation of GRK2 in G2 might also restrain the level of cyclin B1 upregulation at G2/M transition. Cyclin B1 levels are regulated via periodic transcription and dynamic shuttling into and out of the nucleus. Both transcription and influx into the nucleus peak in late G2, when accumulated cyclin B1 can bind and activate CDK1 for mitosis onset. As part of the mechanisms that counteract a constitutive nuclear import and premature CDK1 activation, cyclin B is retained in the cytoplasm by means of its association with Patched1 [163]. Interestingly, GRK2 interacts with Patched1 and inhibits cyclin B1–Patched 1 interaction, allowing for cyclin B1 nuclear accumulation and G2/M transition, whereas GRK2 knockdown in cells and zebrafish results in cell cycle arrest in G2/M stage with accumulation of the mitotic marker pH3 pointing to mitosis failure [150].

In contrast to the catalytic function of GRK2 in Mst2 regulation and centrosome disjunction, regulation of cyclin B1-mediated cell proliferation involves a physical interaction of GRK2 with Patched1. Therefore, both scaffold and catalytic functions of GRK2 coexist in the control of cell cycle during G2/M progression. This complexity would add to the multifactorial role of GRK2 in other transitions of the cell cycle, which are not equally exhibited in the proliferation status of tissues and organs throughout the body, and may help to understand the marked discrepancies in phenotypes in animal models with GRK2 deficiency (global vs. tissue-specific, developmental vs. adult-inducible, total vs. partial). While partial decrease of GRK2 dosage/activity might be beneficial to treat some pathological conditions (e.g., cardiovascular conditions or metabolic syndrome, [18]), sharp and continued reduction of its activity may cause alterations in tissue/organ proliferation. For instance, mice engineered to globally express an shGRK2 transgene to knockdown the kinase without compromising embryonic development, were smaller in size with lower body weight in the adulthood [164] and exhibited renal hypotrophy with reduced glomerular filtration, reduced number of mature glomeruli, and increased hypertension due to overactivation of the renin–angiotensin system [165]. While hypertension is completely reversed by AT1R blockers, defective glomerular filtration is not, suggesting that non-hemodynamic functions related to mesangial proliferation and hypertrophy are altered in the absence of GRK2.

### Emerging roles of the GRK2–HDAC6 signaling axis

The reported functional interaction between GRK2 and HDAC6 (histone deacetylase 6) constitutes a very interesting and prototypic example of stimulus- and context-dependent GRK2 multifunctionality, with potentially relevant implications in a variety of physiological processes and pathological conditions, including epithelial cell motility, viral infection or breast cancer progression.

GRK2 and HDAC6 were first identified as signaling partners in the context of cell migration. GRK2 had been shown to play a negative modulatory role in chemokine receptor-mediated immune cell migration, consistent with its canonical role in agonist-induced GPCR desensitization [166–170]. However, the role of GRK2 in cell motility is more complex, and involves the modulation of diverse phases of this process in a stimulus- and a cell type-specific manner [20]. Strikingly, GRK2 was shown to play a positive role in the migration of epithelial cells and fibroblasts in response to the stimulation of either the sphingosine-1-phosphate (S1P)/S1P1R (a GPCR family member) or fibronectin-mediated integrin activation [64]. In response to these stimuli, GRK2 transiently interacts with the ARF GTPase-activating protein GIT1 (Mammalian G protein-coupled receptor kinase InTeractor 1) [171] at the cell leading edge. Interestingly, the GRK2/GIT1 interaction was dynamically modulated by the GRK2 phosphorylation status by c-Src or MAPK. Rapid migratory stimuli-triggered GRK2 tyrosine phosphorylation in epithelial cells favors GRK2/GIT1 interaction, and thus localized Rac1/PAK/MEK/ERK activation [64]. In turn, GRK2 phosphorylation at S670 by ERK1/2 decreases its ability to associate with GIT1, allowing dynamic assembly/disassembly of these actin cytoskeleton modulatory scaffolds [20, 64, 172].

Importantly, such GRK2 S670 phosphorylation event taking place in epithelial cells in response to the presence of serum, EGF, or other activators of the MAPK pathway, not only disrupts the GRK2/GIT1 signalosome but also promotes the functional interaction of GRK2 with the cytosolic histone deacetylase type II protein HDAC6. Phosphorylation of HDAC6 by GRK2 at residues S1060, 1062, and 1069 promotes increased deacetylation of alpha-tubulin, a well-known HDAC6 target [173], consequently allowing GRK2 to modulate microtubule (MT) dynamics, and thus cell motility and spreading [71].

It is important to note that S670 phosphorylation at the C terminus of the kinase is relevant for the triggering of the GRK2/HDAC6 module not only by favoring GRK2 dissociation from competing cellular partners such as GPCRs [69] or GIT-1 [64], likely by altering electrostatic interactions involved in their binding, but also by fostering a competent conformation at the GRK2 active site that allows phosphorylation of HDAC6 [71]. Since it has been proposed that an allosteric interaction occurs between the C-tail PH domain and the RH domain of GRK2, it is tempting to suggest that alterations initiated by the phosphorylation at S670 would be conveyed to the catalytic domain (Fig. 1), affecting its conformation and promoting a switch in substrate specificity.

Therefore, GRK2 phosphorylation events are instrumental to locally and rapidly switch the repertoire of GRK2 substrates and cellular partners (see Fig. 2), thus specifying the preferred targets of this multifunctional kinase in a dynamic and context-specific way [20, 71]. In the framework of epithelial cell migration, sequential changes in the tyrosine and S670 phosphorylation status of GRK2 would allow the coordinated participation of GRK2/GIT1 and GRK2/HDAC6 signalosomes in several steps of the motility process.

These data suggested that the GRK2/HDAC6 signaling module might be particularly operative and relevant in pathological contexts characterized by increased expression of these proteins and/or high activation of ERK1/2 transduction cascades. On the other hand, they raised the question of whether GRK2-mediated HDAC6 phosphorylation may also affect the activity of the deacetylase toward additional cellular targets [174]. Consistent with this notion, recent data have emphasized a relevant role for the GRK2/HDAC6 axis in breast cancer [12, 19, 111]. Notably, GRK2 protein levels are increased in a variety of breast cancer cell lines, in spontaneous mammary tumors developing in MMTV-HER2 transgenic mice, and in a significant proportion of two independent groups of invasive ductal carcinoma patients [111], in which luminal subtypes predominate.

Importantly, such GRK2 upregulation in breast cancer cells would concur with that of HDAC6, known to be overexpressed in these tumor contexts [111, 175, 176], as well as with increased phosphorylation of GRK2 on S670 [111] as a consequence of the hyperactivation of ERK1/2 downstream of hyperactivated EGFR, HER2, estrogen receptors, or alterations in the Ras pathway often found in both luminal and basal breast cancer contexts. Such conditions would favor the switch of the GRK2/HDAC6 axis.

In fact, this module appears to play a relevant role in fostering proliferation and survival of transformed breast cells, by mechanisms involving GRK2-mediated HDAC6 activation and the modulation of the acetylation status of a newly identified HDAC6 substrate, the prolyl isomerase Pin1, a fundamental cancer driving node also described to be present in such pathological conditions [177].

Enhanced GRK2 protein in breast cells strengthens EGF or heregulin-triggered mitogenic (ERK1/2) and survival (AKT) cascades, fosters proliferation, survival and anchorage-independent growth of luminal MCF7 or MDA-MB-231 basal cancer cells, and increases tumor growth in vivo in xenograft and orthotopic mouse models [111]. On the contrary, silencing GRK2 expression has opposite effects in both luminal and basal breast cancer cells [111, 178]. The molecular mechanisms underlying such positive effects of GRK2 on oncogenic hallmarks appear to involve the modulation of the HDAC6/Pin1 axis downstream of EGFR activation. On the one hand, given that both GRK2 and HDAC6 can interact with the EGFR [2, 21, 60, 179, 180], that EGF triggers GRK2 phosphorylation at S670 [2] and that HDAC6 potentiates EGF signaling by lessening EGFR internalization [179, 180], it is tempting to suggest that phosphoS670-GRK2-enhanced HDAC6 deacetylase activity would allow sustained EGFR signaling by downregulating EGFR internalization [179]. On the other hand, EGF stimulation triggers rapid HDAC6 and Pin1 association and de-acetylation of Pin1 at K46, a process fostered by increased GRK2 expression [111]. In turn, Pin1 deacetylation augments its stability, its prolyl isomerase activity, and the interaction with key downstream oncogenic mediators. Acetylation–deacetylation-mediated variations in charge at K46 might alter the inter-domain linker folding in Pin1, leading to allosteric conformational changes that enhance Pin1 ligand affinity and catalytic activity [111]. Consistent with a relevant pathological role for GRK2-medited changes in Pin1 acetylation status, GRK2 expression and Pin1 levels and de-acetylation status correlate in both cell models and in samples from breast cancer patients [111].

In summary, the EGFR–GRK2–HDAC6–Pin1 axis emerges as a relevant signature and potential therapeutic target in breast cancer. Several key questions remain to be addressed. First, does the GRK2/HDAC6 module promote changes in the acetylation status and activity of other players in breast tumor progression? Do other stimuli also trigger this axis? Are there additional cellular substrates for GRK2 that depend on its prior phosphorylation at S670? These results also raise the possibility that this GRK2/HDAC6 module could also be relevant in other cell types and pathological conditions in which these molecules are known to be involved. In this regard, our unpublished observations suggested a role for the GRK2/HDAC6/Pin1 axis in certain cardiac pathological conditions (Penela et al. in preparation).

Other potentially interesting context is viral infection. Histone deacetylase 6 (HDAC6) plays both enzymatic and scaffold roles in diverse cellular processes including autophagy, pathogen sensing, and cellular cargo movement, related to infection and innate immunity. HDAC6-mediated deacetylation reduces microtubule stability and impairs kinesin and dynein-dependent transport of various cargoes, and such movement mechanisms are co-opted by pathogens, including viruses and intracellular bacteria [181–183]. HDAC6 has also been related to the regulation of viral fusion, entry, nuclear trafficking, replication, assembly, and egress [182]. Interestingly, GRK2 has been shown to support infection of several flaviviruses, such as yellow fever virus (YFV), dengue virus (DENV), and Hepatitis C virus (HCV) [184], and a recent report using quantitative phosphoproteomics in A549 human lung epithelial cells indicates that GRK2 is crucially involved in the initial steps of Influenza A virus (IAV) infection [185]. GRK2 pharmacological inhibitors or siRNA kinase silencing decreases IAV uncoating and limits viral replication in primary human airway epithelial cultures and in established mice models [185]. Of note, GRK2 kinase activity is required for IAV uncoating and the role of this kinase requires a cellular target and is independent of beta-arrestin functions.

IAV harnesses the host endocytic machinery to enter the cell and traffic to reach the replication site in the nucleus. Interaction of viral hemagglutinin with plasma membrane proteins has been suggested to induce clustering and activation of receptor tyrosine kinases such as the EGFR, leading to stimulation of PI3K and ERK1/2 downstream cascades and endocytosis, all processes relevant to engulfing of viral particles and for endosomal acidification leading to viral fusion [185, 186]. Importantly, IAV infection induced EGFR-dependent GRK2 phosphorylation at tyrosine residues and its translocation to EGFR-containing plasma membrane clusters [185]. Given that HDAC6 is recruited to viral fusion sites and suggested to allow efficient un-coating by complex mechanisms involving the aggresome machinery [187], and considering previous reports showing EGFR/GRK2/HDAC6 functional interactions [58, 60, 71, 111], it is tempting to suggest that this module may underlie GRK2 pro-viral function. Whether GRK2–HDAC6 axis is controlling these processes, it is a matter of further research. However, the relationship might not be straightforward. The phosphoproteomic analysis did not detect GRK2 or HDAC6 serine phosphopeptides, although this could be due to transient phosphorylation or protein abundance issues. It is also possible that, in this context, GRK2 might be alternatively activated via tyrosine phosphorylation [58, 60] downstream IAV/EGFR stimulation. However, α-tubulin deacetylation is not required for viral uncoating [187]. Therefore, further investigation should address whether the acetylation status of other targets downstream the GRK2/HDAC6 axis and/or phosphorylation of additional GRK2 substrates upon IAV/EGFR-mediated GRK2 tyrosine phosphorylation, and translocation are involved in the role of this multifunctional kinase in viral infection.
